# A Genetic Risk Score Combining Ten Psoriasis Risk Loci Improves Disease Prediction

**DOI:** 10.1371/journal.pone.0019454

**Published:** 2011-04-29

**Authors:** Haoyan Chen, Annie Poon, Celestine Yeung, Cynthia Helms, Jennifer Pons, Anne M. Bowcock, Pui-Yan Kwok, Wilson Liao

**Affiliations:** 1 Department of Dermatology, University of California San Francisco, San Francisco, California, United States of America; 2 Cardiovascular Research Institute, University of California San Francisco, San Francisco, California, United States of America; 3 Institute for Human Genetics, University of California San Francisco, San Francisco, California, United States of America; 4 Division of Human Genetics, Department of Genetics, Washington University School of Medicine, St. Louis, Missouri, United States of America; Aarhus University, Denmark

## Abstract

Psoriasis is a chronic, immune-mediated skin disease affecting 2–3% of Caucasians. Recent genetic association studies have identified multiple psoriasis risk loci; however, most of these loci contribute only modestly to disease risk. In this study, we investigated whether a genetic risk score (GRS) combining multiple loci could improve psoriasis prediction. Two approaches were used: a simple risk alleles count (cGRS) and a weighted (wGRS) approach. Ten psoriasis risk SNPs were genotyped in 2815 case-control samples and 858 family samples. We found that the total number of risk alleles in the cases was significantly higher than in controls, mean 13.16 (SD 1.7) versus 12.09 (SD 1.8), p = 4.577×10^−40^. The wGRS captured considerably more risk than any SNP considered alone, with a psoriasis OR for high-low wGRS quartiles of 10.55 (95% CI 7.63–14.57), p = 2.010×10^−65^. To compare the discriminatory ability of the GRS models, receiver operating characteristic curves were used to calculate the area under the curve (AUC). The AUC for wGRS was significantly greater than for cGRS (72.0% versus 66.5%, p = 2.13×10^−8^). Additionally, the AUC for *HLA-C* alone (rs10484554) was equivalent to the AUC for all nine other risk loci combined (66.2% versus 63.8%, p = 0.18), highlighting the dominance of *HLA-C* as a risk locus. Logistic regression revealed that the wGRS was significantly associated with two subphenotypes of psoriasis, age of onset (p = 4.91×10^−6^) and family history (p = 0.020). Using a liability threshold model, we estimated that the 10 risk loci account for only11.6% of the genetic variance in psoriasis. In summary, we found that a GRS combining 10 psoriasis risk loci captured significantly more risk than any individual SNP and was associated with early onset of disease and a positive family history. Notably, only a small fraction of psoriasis heritability is captured by the common risk variants identified to date.

## Introduction

Psoriasis is a chronic, immune mediated skin disease, which affects approximately 2–3% of Caucasians [1]. A number of studies have supported the finding that genetic predisposition has a critical role in the development of psoriasis [2], [3]. Identification of psoriasis susceptibility genes is the key to understanding its pathogenesis and improving its treatment [2]. By using linkage analysis and recent genome-wide association studies (GWAS), several loci have been identified as risk factors for the development of psoriasis [4]–[13]. Among them, the *HLA-Cw6* allele has been studied for many years and been known to confer the greatest genetic effect in Caucasians, particularly for early onset cases [14]. However, the penetrance of *HLA-Cw6* was estimated to be only 10%, suggesting *HLA-Cw6* alone was insufficient to explain the disease [15]. Additional non-MHC susceptibility loci have been identified; however, when examined individually each of these loci only confer modest disease risk and is of limited utility in disease prediction. Previous studies have shown that combining multiple loci with modest effects into a global genetic risk score (GRS) might improve identification of persons who are at risk for common complex diseases [16]–[18]. In this study, we examine the discriminatory and predictive ability of a psoriasis GRS and investigate the relationship between the GRS and several psoriasis subphenotypes such as age of onset, family history of psoriasis, type of psoriasis (plaque vs. guttate), and psoriatic arthritis. In addition, we evaluate possible pair-wise genetic interactions and estimate the proportion of genetic variance explained by the known risk alleles.

## Methods

### Subjects

The study population and source are shown in Table 1. All participants provided written informed consent with approval from either the Committee on Human Research at the University of California, San Francisco or the Human Research Protection Office at Washington University, St. Louis. In total, the case-control cohort consisted of 731 unrelated cases and 2084 unrelated controls of European ancestry. A total of 1104 case-control samples (642 from University of California San Francisco (UCSF) and 462 from Washington University in St. Louis (WashU)) were genotyped by the Illumina GoldenGate assay. 1711 control samples obtained from studies 66 and 67 of illumina iControlDB were genotyped on Human Hap550 BeadChip. Psoriasis cases were 50.4% female, had mean age of onset 24.6 yrs (SD 15.5), and had a positive family history of psoriasis, defined as at least one affected first-degree relative, of 76.7%. Family samples from WashU consisted of 281 families with at least 1 sibling diagnosed with psoriasis. This sample included 225 unaffected subjects and 633 affected subjects (among the 633 affected subjects, 261 probands were also included as cases in the case-control cohort). Almost all the families were nuclear, two generation families consisting of children and their parents, and thus nearly all the subjects were first degree relatives. The mean number of affected subjects per family was 2.32 (0.71), and the mean number of unaffected subjects per family was 1.24 (0.44).

**Table 1:** Case-control population, family subjects and source.

Case-control samples	N (Psoriasis Case)	N (Control)	Genotyping platform
UCSF and WashU	731	373	Illumina Goldengate Assay
Illumina's iControlDB	NA	1711	Hapmap 550k BeadChip
Total	731	2084	_

Family subjects	N (Affected family members)	N (unaffected family members)	Genotyping platform
WashU	633*	225†	Sequenom iPLEX

*Among the 633 affected family subjects, 261 probands were also included as cases (subset of 731) in case-control samples.

† Note: The 225 unaffected family subjects were not included in generating the risk allele odds ratios.

UCSF: University of California San Francisco; WashU: Washington University in St. Louis

Illumina's iControlDB: an online database of genotype and phenotype data from individuals that can be used as controls in association studies. These data are generated from Illumina genotyping products.

### Selection of genetic risk factors and quality control

We selected the psoriasis risk alleles from GWAS demonstrating p<5×10^−7^ and from large cohort studies with evidence of replication at p<0.05 in at least one independent study. In total, 11 SNPs shown to be associated with psoriasis in previous GWAS or large cohort studies were selected for analysis (Table S1). For case-control samples, all 11 SNPs were successfully genotyped by the Illumina GoldenGate assay. All 1104 samples passed a quality control check (Illumina GenTrain score > 0.5, MAF>0.01, Hardy-Weinberg Equilibrium, p>0.0001). We further removed the suspected duplicates using an Identity-By-State and Identity-By-Descent check (15 subjects from cases and 3 from controls). Family samples were genotyped using the iPLEX chemistry on the Sequenom platform according to the manufacturer's instructions (Sequenom, San Diego, CA).

Prior to merging, the iControlDB data were filtered for individuals with <90% genotyping and SNPs with <90% genotyping, MAF <1%, or HWE p-value <0.00001. Three SNPs genotyped by the Illumina GoldenGate assay but not present in the Human Hap550 BeadChip were imputed by the program IMPUTE2 [19], [20], using both HapMap (NCBI Build 36 (db126b)) CEU data and 1000 Genomes as a reference haplotype set. Two SNPs (rs17728338, rs6125829) showed high imputation confidence (98.7% and 99.5%). One SNP rs597980 with low imputation confidence (79.9%) was excluded from further analysis. Two SNPs (rs11209026 and rs10484554) had missing genotyping data (17/1711 and 36/1711, respectively) in the iControlDB dataset. We use IMPUTE2 to impute the missing genotypes for the two SNPs. Sixteen samples for rs11209026 and thirty-two samples for rs10484554 were accurately imputed.

To correct for stratification of northwest vs. southeast European ancestry, cases and controls were genotyped for 46 ancestry informative markers (AIMs) which comprised a subset of those previously reported [21]. All of these 46 SNPs were also present on the Hapmap 550k platform used by iControlDB. Forty AIMs passed the quality control criteria (GenTrain score >0.5, MAF>0.01 and HWE, P>0.0001). We evaluated these AIMs on a test cohort of individuals from geographically distinct areas of Europe and validated their ability to distinguish northwest–southeast ancestry (data not shown). Principal component analysis as implemented in EIGENSTRAT was used to analyze the samples [22]. ANOVA statistics for population differences along the eigenvectors demonstrated that cases and controls did not differ on the first three principal components. However, there was a significant effect of case status on the fourth principal component (p = 0.01). No significant difference between cases and controls was observed for any of the remaining principal components. The first four principal components were used as covariates for ancestry adjustment.

### Genetic risk score computation

Two approaches were used to calculate the GRS: a simple risk alleles count method (count GRS, cGRS) and a weighted method (weighted GRS, wGRS).The wGRS weights each risk allele by the logarithm odds ratio (Log(OR)) for that allele in our dataset. Thus, the wGRS is a linear combination of the number of risk alleles weighted by the Log(OR) as coefficients. For example, two IL12B risk alleles contribute 2× Log(1.59)  = 0.928 to the wGRS. For a protective SNP, the risk alleles are the major alleles. In order to reduce the bias caused by missing data, we did not include any subjects who had one or more missing genotypes in generating the GRS. Because of the extremely low rate of missing data to start with, only 7 cases and 89 controls were removed by this process.

### Percentage of genetic variance explained

The percentage of genetic variance was estimated under a liability threshold model [23], [24]. This model assumes that psoriasis has an underlying liability score which is normally distributed with mean 0 and variance 1. We assumed a psoriasis prevalence of 2.5% in the general population. To calculate the threshold for each genotype, we used allele frequencies from controls and an effect size corresponding to the OR from our analysis.

### Subphenotype definitions

Determination of plaque versus guttate psoriasis was made by a dermatologist. Age of psoriasis onset was self-reported by subjects. A positive family history was defined as at least one first degree family member affected with psoriasis. The presence of psoriatic arthritis was defined as subjects reporting psoriatic arthritis diagnosed by a rheumatologist or dermatologist.

### Statistical Analysis

An R software package (http://www.r-project.org/) was used for logistic regression analysis, odds ratio estimation and comparison of ROC curves. The Hardy-Weinberg equilibrium test, pair-wise interactions test and the association test for individual loci were performed by PLINK [25].The non-parametric Mann-Whitney test was used to compare the mean number of risk alleles in cases-control population and the mean wGRS in family samples. Correlation between the wGRS of affected and unaffected family members within families was measured using a Spearman correlation test. Cochran-Armitage trend test was used to evaluate association of wGRS quartiles with psoriasis. All the association tests were adjusted by gender and first four principal components.

## Results

### Association between genetic risk alleles and psoriasis

Ten SNPs previously confirmed as psoriasis susceptibility loci were evaluated in this study. Each SNP was in Hardy-Weinberg equilibrium (P≥0.05) in the control group. The results for each of the ten risk alleles with the risk of psoriasis are shown in Table 2, along with their adjusted odds ratios (ORs). Calculation of ORs was performed using only case-control subjects. Associations range from OR = 3.07 (95% CI 2.61–3.61, 9.67×10^−42^) for the SNP *of HLA-C* to OR = 1.06 (95% CI 0.89–1.25, p = 0.5288) for *IL13*. All the ORs are in the same direction for the risk alleles from published discovery studies.

**Table 2:** Ten SNPs used to calculate the psoriasis genetic risk score in case-control samples.

Chr	Gene	SNP	Allelesrisk/nonrisk	Risk alleles frequency		OR*	95% CI*	P-add*
				Case	Control			
1	*IL23R*	rs11209026	G/A	0.961	0.938	1.61	1.16–2.24	0.0044
1	*LCE3C/3D*	rs4112788	C/T	0.701	0.635	1.37	1.18–1.58	2.31E-05
5	*IL13*	rs20541	C/T	0.813	0.806	1.06	0.89–1.25	0.5228
5	*TNIP1/ANXA6*	rs17728338	A/G	0.109	0.061	1.94	1.54–2.45	2.96E-08
5	*IL12B*	rs3213094	G/A	0.853	0.784	1.59	1.33–1.91	5.80E-07
6	*CDKAL1*	rs6908425	C/T	0.823	0.792	1.22	1.03–1.45	0.0223
6	*HLA-C*	rs10484554	T/C	0.351	0.158	3.07	2.61–3.61	9.67E-42
6	*TNFAIP3*	rs610604	C/T	0.364	0.336	1.12	0.98–1.29	0.1083
12	*IL23A/STAT2*	rs2066808	T/C	0.955	0.920	1.82	1.34–2.45	0.0001
20	*ZNF313*	rs6125829	G/T	0.646	0.616	1.13	0.98–1.30	0.0843

*P-value and ORs were adjusted for 4 principal components and gender.

Chr: Chromosome

SNP: Single nucleotide polymorphism

OR: Odds ratio

CI: Confidence intervals

P-add: P value under additive model

### Genetic risk score association

**Figure 1:**
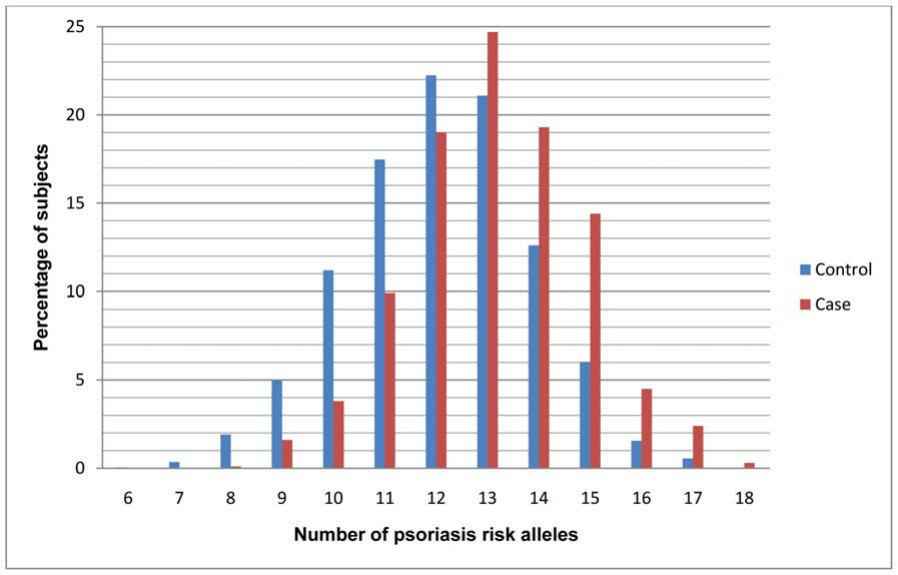
Distribution of the number of psoriasis risk alleles in cases (red) and controls (blue).

**Figure 2:**
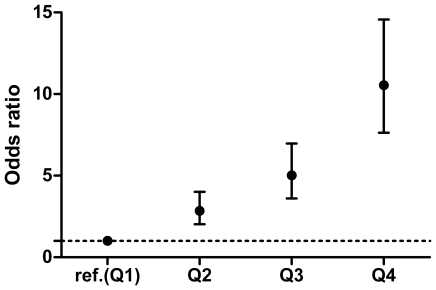
Psoriasis odds ratios of weighted genetic risk score quartiles relative to the first quartile. vertical bars correspond to 95% confidence intervals. Ref.: reference. Q: quartile.

To evaluate the cumulative effects of the ten highly replicated psoriasis risk alleles, we developed a GRS by using either a simple risk alleles count (cGRS) or a weighted (wGRS) approach. Figure 1 shows the distribution of number of psoriasis risk alleles in both cases and controls, with a shift towards a higher number of risk alleles in the cases. The mean number of risk alleles in the cases is 13.16 (SD 1.7) and the mean number of risk alleles in the controls is 12.09 (SD 1.8), with a p = 4.577×10^−40^ by using the Mann-Whitney test. To estimate the total amount of risk captured by the genetic risk score, we calculated the odds ratios according to wGRS quartile (Figure 2). There is a significant increase in psoriasis ORs with increasing wGRS quartile, using the first quartile as the reference group. The psoriasis OR, adjusted for intra-European ancestry and gender, for high-low wGRS quartiles is 10.55 (95% CI 7.63–14.57), with a trend p = 2.010×10^−65^.

**Figure 3:**
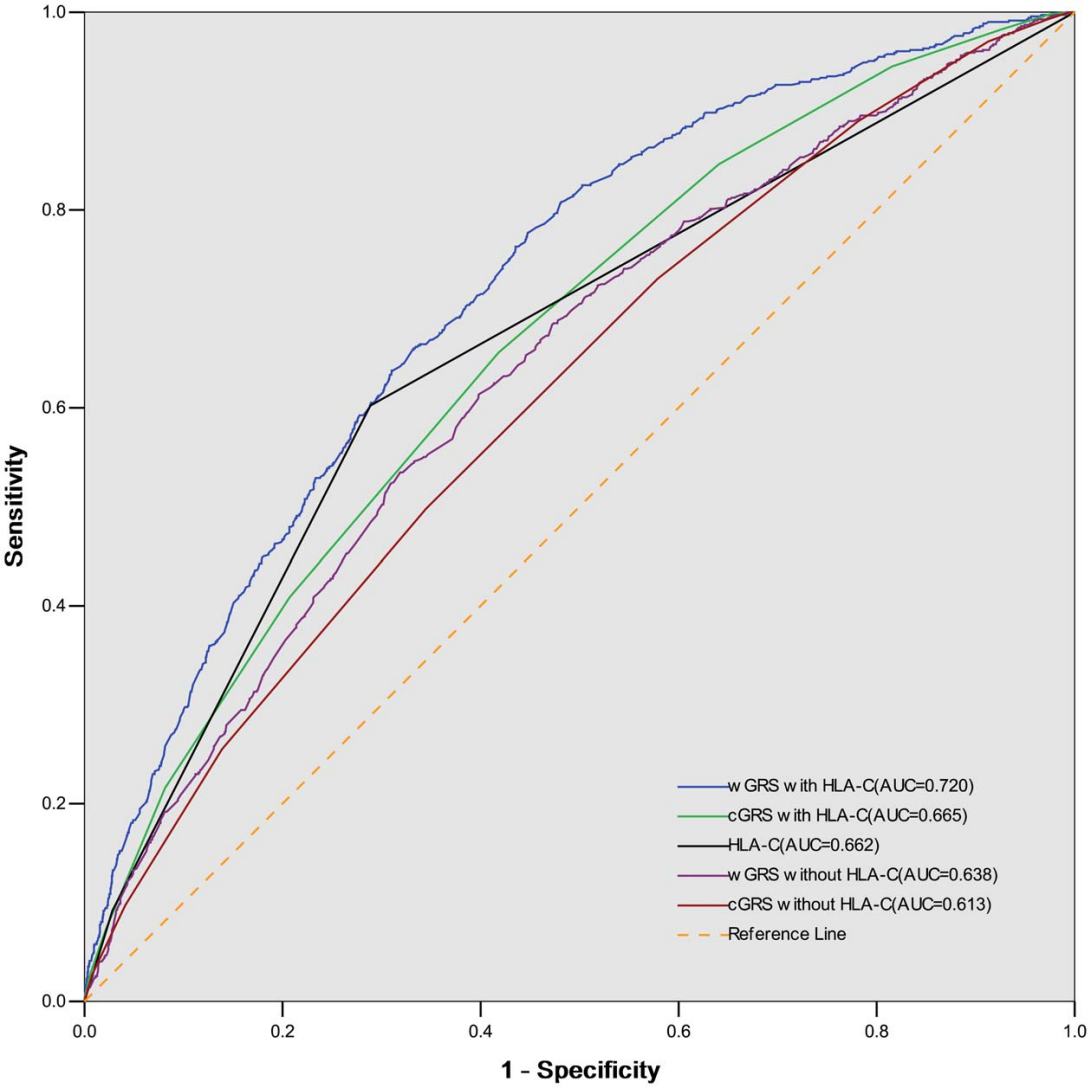
Receiver-operating characteristic curves for models predicting diagnosis psoriasis. The AUC for wGRS is significantly greater than AUC for cGRS, p = 2.13×10^−8^. The AUC for HLA-C alone (rs10484554) is not statistically different than the AUC for cGRS, and no worse than the AUC for the wGRS with HLA-C excluded, p = 0.18. AUC: area under curve. wGRS: weighted genetic risk score. cGRS: counted genetic risk score.

We next measured and compared the discriminative power attributable to the GRS by plotting receiver operating characteristic (ROC) curves and calculating the area under the curve (AUC) for the case-control samples. Our result shows that the wGRS is a significantly better predictor than cGRS (Figure 3). The AUC for wGRS is 72.0% (95%CI 69.9% –74.1%) versus 66.5% (95%CI 64.2% –68.8%) for cGRS, p = 2.13×10^−8^. Interestingly, the AUC for *HLA-C* alone (rs10484554) is 66.2%, which is not statistically different than the AUC for cGRS (66.5%, p = 0.83), and even slightly better than the AUC for the wGRS with HLA-C excluded(63.8% p = 0.18).

### Associations with sub-phenotypes

Logistic regression was used to examine the relationship between the wGRS and psoriasis subphenotypes, including age of onset, family history of psoriasis, type of psoriasis, and psoriatic arthritis. As shown in Table 3, two significant associations were found between the wGRS and age of onset (p = 4.91×10^−6^)and family history of psoriasis(p = 0.020), with corresponding ORs of 0.559(0.433–0.714) and 1.34(1.050–1.723). To further examine whether a positive family history of psoriasis is associated with a higher wGRS, we calculated the wGRS for affected and unaffected subjects from psoriasis families, and compared the results to the wGRS for our unrelated case-control samples. To eliminate the impact of unequal contribution due to family size, the mean wGRS of affected subjects and unaffected subjects within each family was first computed, resulting in a “wGRS_family, affected_” and “wGRS_family, unaffected_.” We found that the mean wGRS_family, affected_ in affected family subjects was significantly higher than mean wGRS in cases without family history (4.92 versus 4.74, p = 0.014). We also observed the mean wGRS_family, unaffected_ was slightly greater than in healthy controls (4.29 versus 4.20, p = 0.071). We also investigated the nature of intra-family vs inter-family variation by plotting wGRS_family, affected_ against wGRS_family, unaffected_ in 180 families where data were available for both affected and unaffected members. While significant variation in the wGRS is seen between families, a strong positive correlation is seen between wGRS of unaffected subjects and affected subjects within each family (R = 0.299, p = 4.58×10^−5^, Figure S1).

**Table 3:** Association of wGRS with psoriasis subphenotypes.

	N	Reference phenotype	Test phenotype	OR* (95% CI)	p-value*
Family history	514	No	Yes (≥1 first-degree relatives affected)	1.340(1.050–1.723)	0.020
Age onset	537	≤30 yrs	>30 yrs	0.559(0.433–0.714)	4.91E-06
Psoriatic arthritis	560	No	Yes	0.849(0.688–1.043)	0.121
Psoriasis type	557	plaque	guttate	1.342(0.923–1.851)	0.072

*P-value and ORs were adjusted for first 4 principal components and gender.

OR: Odds ratio.

95%CI: 95% confidence intervals.

### Gene-gene Interactions

We also investigated all potential pair-wise interactions of the 10 risk alleles in both psoriasis cases versus controls and in case-only analysis for each subphenotype. However, we did not find any evidence of interactions between each SNP after Bonferroni correction (data not shown).

### The percentage of genetic variance explained

To estimate the percent of variance explained by each of the psoriasis risk alleles, a previously described liability threshold model was used [26], [27].Our data show that *HLA-C*, *IL12B* and *TNIP1/ANXA6* were estimated to account for 6.7%, 1.3% and 1.0% of the genetic variance, whereas the remaining loci each accounted for less than 1% of the genetic variance (Table 4). Together, the 10 psoriasis risk loci were able to explain an estimated 11.6% of the total genetic susceptibility to psoriasis.

**Table 4:** The percentage of genetic variance explained by selected SNPs.

Chr	Gene	SNP	Additive genetic variance explained*
1	*IL23R*	rs11209026	0.0045
1	*LCE3C/3D del*	rs4112788	0.0083
5	*IL13*	rs20541	0.0002
5	*TNIP1/ANXA6*	rs17728338	0.0101
5	*IL12B*	rs3213094	0.0127
6	*CDKAL1*	rs6908425	0.0023
6	*HLA-C*	rs10484554	0.0666
6	*TNFAIP3*	rs610604	0.0011
12	*IL23A/STAT2*	rs2066808	0.0089
20	*ZNF313*	rs6125829	0.0013

*The percentage of variance explained was estimated under liability threshold model.

Totally, the selected 10 SNPs could explain11.6% of genetic variance.

SNP: Single nucleotide polymorphism.

Chr: chromosome.

## Discussion

In this study, we developed a psoriasis genetic risk score (GRS) which included 10 highly replicated loci from recent GWAS and large cohort studies. We found that a weighted GRS, which accounts for the odds ratio of each allele, is a better discriminator of cases and controls than a simple count GRS. This agrees with the results of Piccolo et al [26] who also found that a weighted GRS could capture considerably more genetic risk compared to a cGRS. Our weighted GRS was more associated with risk of psoriasis than any single SNP alone, with persons in the highest wGRS quartile having a greater than 10-fold increased risk of psoriasis compared to persons in the lowest quartile.

Of the 10 SNPs evaluated in this study, the strongest signal was found at the *HLA-C* locus at rs10484554, for which there was a 206% elevated risk of psoriasis with each risk allele. Notably, the predictive capability for this single SNP was as good as that of the other 9 non-MHC loci combined (Figure 3). Furthermore, our study may actually underestimate the true effect of *HLA-Cw6* because this allele has been shown to be more strongly associated with psoriasis than its tag SNP rs10484554 used in this study [27]. The strong effect of this single locus on disease susceptibility stands in contrast to other common diseases such as Type 2 diabetes, coronary artery disease and prostate cancer, in which the risk is more evenly distributed amongst multiple alleles [16], [28], [29]. Thus, the genetic architecture underlying common complex diseases may vary according to the disease. The 9 additional non-MHC SNPs had a more modest effect on the risk of psoriasis, with each allele conferring an elevated risk of 94% or lower. While these alleles have little predictive power individually [30], their combined addition to the *HLA-C* allele resulted in a composite wGRS that displayed a slightly enhanced ability to discriminate between people with and without psoriasis.

With regard to sub-phenotypes, our data show that the weighted genetic risk score was higher amongst cases with an earlier-onset of psoriasis and with a family history of disease. This may largely be explained by the observation that the *HLA-C* locus has a large impact on the wGRS, and several studies have previously demonstrated that *HLA-Cw6*-positive patients show an earlier disease onset and greater family aggregation than those without it[5], [31]–[33]. In addition, since a positive family history is a surrogate for genetic predisposition, it is not surprising that a higher GRS was seen in cases with affected first degree relatives. A marginal significance (p = 0.072) was observed between the wGRS and guttate psoriasis. This could be explained by the stronger association of HLA-Cw6 with guttate psoriasis compared to plaque psoriasis [34]. We did not detect an association between the wGRS and PsA. This may be in part due to the small sample sizes of the subgroup analyses. In addition, since the GRS SNPs used in this study were identified based on genetic studies of psoriasis and not PsA per se, lack of association with PsA may simply indicate that the present SNPs do not account for genetic susceptibility to the arthritic component.

The estimated proportion of genetic variation explained by the 10 included SNPs in this study was11.6%, including 6.7% due to *HLA-C*. This suggests that many additional susceptibility loci for psoriasis remain to be discovered. Similar results have been reported in other complex diseases. For example, the proportion of heritability explained by known common SNPs for Crohn's disease, systemic lupus erythematosus and type 2 diabetes was estimated to be around 20%, 15% and 6%, respectively [35]. One hypothesis is that rare variants with higher penetrance, not covered by the present genotyping, might be able to explain the remaining heritability [36]. Searching for such genetic factors are underway thanks to advances in next generation sequencing technologies [15]. Furthermore, although the wGRS is highly significantly associated with psoriasis susceptibility, the predictive capability is seemingly modest (AUC for ROC curve 72.0%). It will be interesting to see whether the discriminatory accuracy can be improved as additional rare variants in psoriasis are included.

Several limitations to this study need to be acknowledged. First, although we captured the most significant psoriasis loci known to date for calculation of the GRS, we did not include all known psoriasis loci. For example, human β defensin (*HBD*) was not evaluated in this study since we were unable to identify a proxy SNP for HBD copy number[37]. Additionally, during the preparation this manuscript, several novel psoriasis loci were identified by very recent genome-wide association studies but not included in this study [38]–[41]. Among them, *TRAF3IP2* was shown to be the most reproducibly associated locus and showed a psoriasis odds ratio of 1.70. However, none of the newly identified loci is estimated to explain more than 1% of the heritability. Inclusion of these additional loci to the SNPs described in our model results in an estimated proportion of heritability explained of 15%. Second, to increase the robustness of our control data set, we included a number of controls from the public iControlDB database. However, we used a panel of European AIMs to correct for potential population stratification. Moreover, when we conducted the association analysis removing the iControlDB subjects, we obtained similar results with respect to the direction of association and magnitude of the odds ratio for each SNP (data not shown). Third, two of the GRS SNPs in the iControlDB dataset were imputed rather than directly genotyped and in two instances we used proxy SNPs for the originally reported variants. Although using imputed or proxy SNPs might lead to less accurate results, we ensured that only SNPs with high imputation confidence >98% and proxy SNPs with r^2^ >0.9 were included in final analysis. Fourth, only Caucasians were included in this study. Thus, the characteristics of the GRS calculated here may not applicable to other ethnic populations. Fifth, our prediction model is based on the same data used to construct the model, which may lead to overfitting and overestimation of the AUC. Finally, this was not a prospectively-designed study, and thus we were unable to truly examine the discriminatory power of these SNPs.

In summary, we found that a GRS combining 10 psoriasis risk loci was significantly associated with an increased risk of psoriasis. The weighted GRS approach increased the power in discriminatory accuracy, compared to the counted GRS and any of the risk alleles considered alone. A higher GRS was associated with early-onset of disease and positive family history. The 10 risk loci account for only 11.6% of the genetic variance in psoriasis, suggesting that additional susceptibility alleles remain to be identified.

## Supporting Information

Figure S1 Correlation among family members. The nature of intra-family vs inter-family variation was investigated by plotting wGRS_family, affected_ against wGRS_family, unaffected_ in each family. A strong positive correlation was observed between wGRS of unaffected subjects and affected subjects within each family(R = 0.299, p = 4.58×10^−5^).
(TIF)

Table S1 SNPs used to estimate psoriasis genetic risk score: references, proxies and imputed SNPs.
(DOC)
