# Supplementary figures and images for: A Genetic Risk Score Combining Ten Psoriasis Risk Loci Improves Disease Prediction
Source: PLoS One. 2011 Apr 29;6(4):e19454. doi: 10.1371/journal.pone.0019454 (PMC3084857; doi:10.1371/journal.pone.0019454)

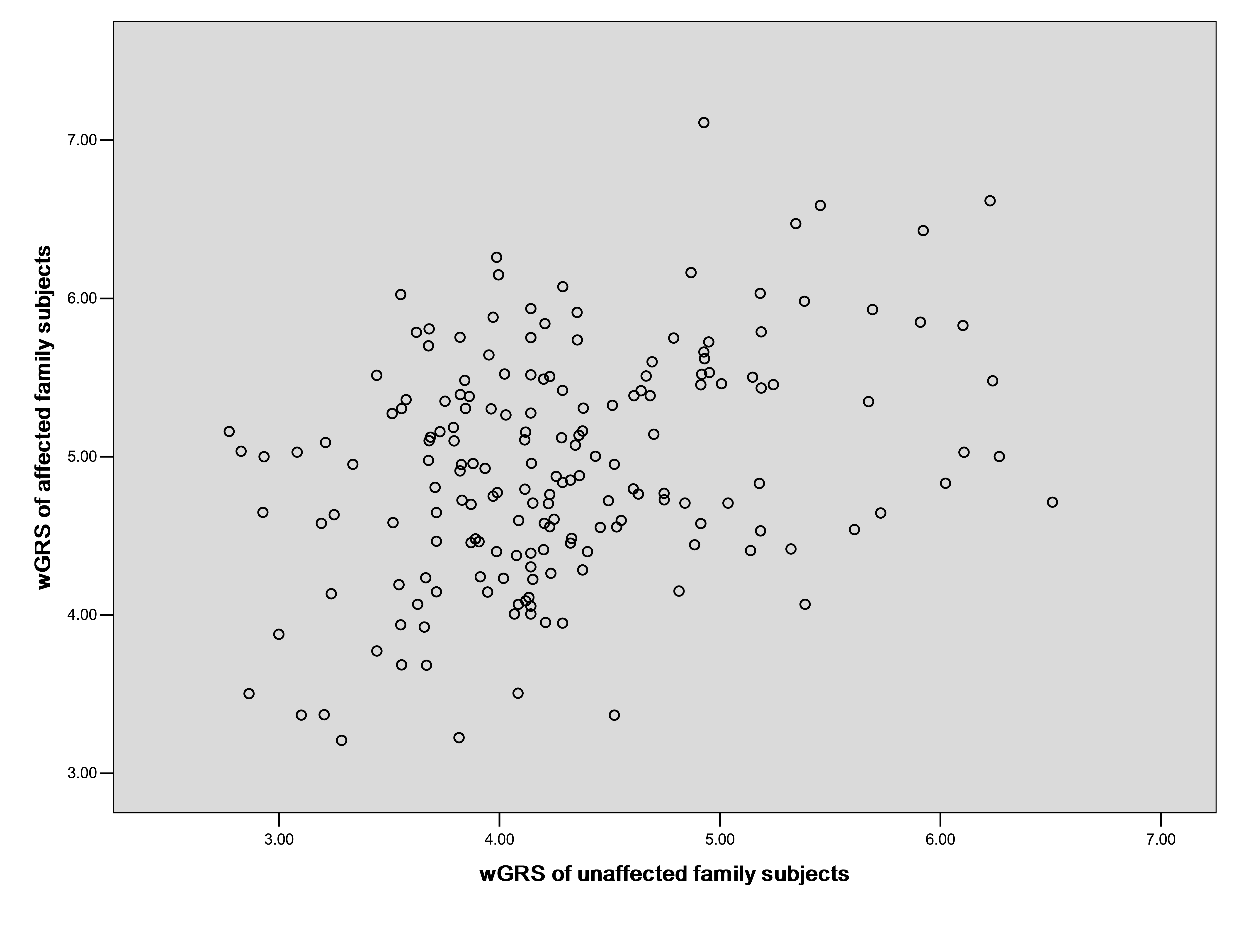

Supplement: Figure S1 — Correlation among family members. The nature of intra-family vs inter-family variation was investigated by plotting wGRSfamily, affected against wGRSfamily, unaffected in each family. A strong positive correlation was observed between wGRS of unaffected subjects and affected subjects within each family(R = 0.299, p = 4.58×10^−5^). (TIF) [file pone.0019454.s001.tif]
