# Supplementary material for: A Genetic Risk Score Combining Ten Psoriasis Risk Loci Improves Disease Prediction
Source: PLoS One. 2011 Apr 29;6(4):e19454. doi: 10.1371/journal.pone.0019454 (PMC3084857; doi:10.1371/journal.pone.0019454)
Supplement: Table S1 — SNPs used to estimate psoriasis genetic risk score: references, proxies and imputed SNPs. (DOC) [file pone.0019454.s002.doc]

**Supplemental Tables**

**Table S1. 11 SNPs used to estimate psoriasis genetic risk score: references, proxies and imputed SNPs**

| **SNP** | **Gene** | **Chr** | **Position** | **SNP reference** | **Proxied allele (r^2^)^Ŧ^** |
| --- | --- | --- | --- | --- | --- |
| rs11209026 | *IL23R* | 1 | 67478546 | Cargill et. al[10] | N/A |
| rs4112788 Ŧ | *LCE3C/3Del* | 1 | 150817900 | de.Cid et .al[8] | *ALCE3C/3D Del* (0.93) |
| rs20541 | *IL13* | 5 | 132023863 | Nair et al[9] | N/A |
| rs17728338* | *TNIP1/ANXA6* | 5 | 150458511 | Nair et. al[9] | N/A |
| rs3213094 | *IL12B* | 5 | 158696755 | Cargill et. al[10] | N/A |
| rs6908425 | *CDKAL1* | 6 | 20836710 | Li et. al[12] | N/A |
| rs10484554Ŧ | *HLA-C* | 6 | 31382534 | Liu et. al[7] | N/A |
| rs610604 | *TNFAIP3* | 6 | 138241110 | Nair et. al[9] | N/A |
| rs2066808 | *IL23A/STAT2* | 12 | 55024240 | Nair et. al[9] | N/A |
| rs597980*^†^ | *ADAM33* | 20 | 3599165 | Li et. al[12] | N/A |
| rs6125829*Ŧ | *ZNF313* | 20 | 48002336 | Capon et. al[11] | rs495337(1.0) |

* SNPs imputed in the iControlDB dataset.

†not included in final analysis due to low imputation accuracy (<.90).

Ŧ The proxy SNPs (r^2^=1 with one another) were genotyped in family samples, rs4085613 (proxy for rs4112788), rs12191877 (proxy for rs10484554), rs495337 (proxy for rs6125829).

SNP: Single nucleotide polymorphism

Chr: chromosome
